# Epidemiology of Bovine Neosporosis in Relation to Socioeconomic, Demographic, and Transmissibility Factors in Dual-Purpose Production Systems in Colombia

**DOI:** 10.3390/epidemiologia5040056

**Published:** 2024-12-19

**Authors:** Cesar A. Murcia-Mono, Sergio Falla-Tapias, Britney K. Cabrera-Ospina, Jahir O. Vargas-Domínguez, William O. Burgos-Paz

**Affiliations:** 1 Semillero de Investigación CIETVET, Programa de Medicina Veterinaria y Zootecnia, Facultad de Medicina Veterinaria y Ciencias Afines, Corporación Universitaria del Huila CORHUILA, Neiva 410010, Colombia; cesar.murciam@corhuila.edu.co (C.A.M.-M.); sergio.falla@corhuila.edu.co (S.F.-T.); bkcabrera20201@corhuila.edu.co (B.K.C.-O.); 2 Centro de Investigación Tibaitatá, Corporación Colombiana de Investigación Agropecuaria AGROSAVIA, Mosquera 250047, Colombia; jovargas@agrosavia.co; 3 Centro de Investigación Turipaná, Corporación Colombiana de Investigación Agropecuaria AGROSAVIA, km 13 vía Montería, Cereté 230550, Colombia

**Keywords:** abortion, biosecurity practices, epidemiological, risk factors

## Abstract

Introduction: Bovine neosporosis represents a significant threat to reproduction and production in livestock systems worldwide. This disease is caused by the protozoan *Neospora caninum*, resulting in abortions of cows and neurological signs in newborn calves. This leads to significant economic losses, decreasing meat and milk production, especially in tropical regions. The infection has an endogenous and exogenous cycle of transmission involving dogs that shed the oocysts, with the highest transmission successes in humid areas. Similarly, there is a lack of knowledge about the epidemiological risk factors and management practices involved in the transmission success in tropical humid regions. Methods: In this sense, a cross-sectional epidemiological survey was conducted on 150 farms from 24 municipalities of the Huila area. A total of 360 cattle were sampled, and information about the production system was collected using a structured poll with 128 questions. Results: In these cattle, 53% (191/360) were positive for antibodies against *Neospora caninum* using ELISA. The logistic regression analysis using the information collected from the poll identified the presence of flooring type, water access, production systems, and feed management as risk factors. Among the protective factors were identified the geographical area, molasses supplementation, and biosecurity practices such as animal separation and access control. Discussion: This study identified for the first time the epidemiological risk factors associated mainly with the exogenous cycle of neosporosis. The present study contributes to the design of intervention strategies oriented to minimize the impact of parasitism in Colombian herds.

## 1. Introduction

Bovine neosporosis caused by *Neospora caninum* has a worldwide distribution, with economic losses of around USD 1.3 billion per year, with a higher impact on cattle in tropical areas [1]. Cows of any gestational period may abort, but most abortions occur between the fourth and sixth month of gestation [2]. Calves acquire the parasite by vertical transmission as the main route of infection in the exogenous and endogenous cycle [3]. Currently, in the absence of vaccines and treatment, the control of neosporosis in cattle includes biosecurity strategies such as test-and-culling of seropositive cattle and reproductive management of positive herds, including the replacement of heifers, embryo transfer, and artificial insemination [4].

This parasitism is widely distributed in South American countries and many parts of Colombia; since its first description in 2001, bovine neosporosis has been confirmed in many Colombian regions, including the highlands of Cundinamarca, Nariño, and Antioquia and the lowlands, such as Córdoba, Amazonas, and Caquetá [5]. However, most of this epidemiological evidence comes from research, with little access to diagnosis and with less routine surveillance by authorities. The main suspicion of parasitism circulation is generated from the occurrence of abortion without retention of the placenta and suggested circulation of infection in other areas [6]. Similarly, little attention has been paid to epidemiological risk factors and environmental conditions favoring transition success in Colombia and other South American countries.

In addition, in Colombian bovine herds, neosporosis is the leading cause of abortion among viral and bacterial diseases: some studies demonstrated that in co-infections between reproductive agents, neosporosis was more commonly found in abortions than other etiologies [7,8]. The success of transmission could be related to the exogenous cycle involving dogs that shed the oocyst to the environment. While infectivity occurs 24–72 h after elimination, it has acquired resistance in certain environments, with the highest survival time in humid environments such as tropical regions, including Colombia [9]. In Colombia, the disease’s properties show a notable difference in rainfall and climate conditions depending on temperature and humidity, such as in altitudes in the mountain range that favor puddle formation and thus the viability of oocysts inside cattle herds [10]. So, extensive and semi-extensive management favors the ingestion of oocysts during grazing, suggesting that cattle could be infected in the early period of life.

After an exogenous transmission cycle, the endogenous cycle maintains the infection inside herds; in this scenario, the longevity of heifers and their offspring contributes to endemism. Similarly, in extensive and semi-extensive systems, the presence of dogs and wild canines contributes to continuous transmission [11]. However, the neosporosis in many areas in Colombia remains unknown, and the factors contributing to transmission are unassessed. So, neosporosis in the country’s southern areas, including the Huila area, is poorly understood. In this scenario, it is crucial to determine the epidemiological status and the local conditions associated with the dynamics of the disease. Thus, this research aims to clarify the epidemiological status of bovine neosporosis in dual-purpose livestock systems and to determine the factors related to the epidemiological dynamics in these production systems to design intervention strategies.

## 2. Materials and Methods

### 2.1. Study Area

A cross-sectional study was carried out on 150 cattle farms in the area of Huila; the municipalities included were Acevedo, Altamira, Aipe, Algeciras, Baraya, Campoalegre, Garzón, Gigante, La Plata, Nátaga, Paicol, Pital, Palermo, Pitalito, Rivera, San Agustín, Suaza, Tesalia, Tarqui, Timaná, Tello, Villavieja, and Yaguará. The project was carried out in collaboration with the Comité de Ganaderos del Huila CGH, the Gobernación del Huila, the Corporación Colombiana de Investigación Agrosavia, and veterinary and technical professionals from the Corporación Universitaria del Huila Corhuila. To guarantee the results’ reliability and the data integrity, the project was endorsed by Agrosavia’s professional ethics and bioethics committee.

### 2.2. Study Population

A total of 360 animals were selected from a total of 150 farms. These cattle complied with the established inclusion requirements. A clinical systemic examination was performed on all the cattle, evaluating physiological constants, weight, body condition, and the anatomical and physiological integrity of the systems. Previously trained veterinary professionals carried out this physical examination. We also collected relevant information on reproductive problems, the socio-economic status of the farm owners, other factors related to the living conditions of the animals, and the records provided by the owners.

### 2.3. Sample Processing and ELISA Tests

Blood samples were collected by venipuncture from the tail vein of all cattle included in the study. These samples were analyzed using the indirect ELISA enzyme immunoassay technique (INgezim® Neospora 3.0 12.NC*.K.1 INGENASA, Madrid, Spain) according to the manufacturer’s recommendations. The ELISA reader (Microplate reader BOECO BMR 100, Hamburg, Germany) was used for reading at 450 nm. Blood was collected in red-capped tubes without anticoagulant and sent to the staff of the Health Biotechnology laboratory of the Corporación Universitaria del Huila Corhuila for diagnosis. Samples were centrifuged at 5000 rpm for 5 min and stored at −20 degrees Celsius. They were then thawed at room temperature for diagnostic analysis. The interpretation of the results was based on the validated results of the controls provided by the kit, where the positivity index (PPI) was calculated, with values lower than 17 as negative and higher than 20 as positive; in the present study, there were no suspicious cases (values between 17 and 20 of PPI).

Seroprevalence was defined as the presence of antibodies against *N. caninum* in the host’s serum. The true prevalence was calculated using the following equation:

$$Prevalence=\frac{Number\;of\;disease\;cases}{Total\;population\;at\;risk}\times 100$$

An epidemiological poll was carried out on management aspects in livestock herds. A total of 128 questions were studied and grouped into different categories: good husbandry practices (GHP), animal health, feeding, reproduction, facilities, veterinary medication, personnel, clinical history, sanitation, transport, traceability, biosecurity, and socio-economic aspects. The aim was to identify sources of risk or protection related to disease. Logistic regression (LR) was used to measure the association between *N. caninum* and the hypothesized causal factors and the interpretation of these associations. OR values greater than 1 (lower confidence interval 95% CI > 1) and with *p* < 0.05 were considered risk factors, while OR values less than 1 95% CI < 1 and with *p* < 0.05 were considered protective factors.

## 3. Results

Of the 360 analyzed samples, 191 tested positive for antibodies against *Neospora caninum*. Table 1 summarizes the seroprevalence of neosporosis stratified by age groups defined in months. The seroprevalence exhibited variation among age groups, ranging from 45.8% in the 15–45-months group to 60.0% in the ≥106-months group.

Furthermore. regional disparities were identified within the area. The southern area showed the highest prevalence at 80% (52/65), followed by the central, western, and northern regions (Table 2). Regarding management aspects. the prevalence of disease exhibited variation according to the interval between deworming (commercial anthelmintics such as Fenbendazole and Ivermectin). In many groups, prevalence was similar, apart from the group whose deworming frequency was 6–12 months (Table 3).

However, the study identified protective and risk factors related to the prevalence of *N. caninum*. The central, northern, and western geographical zones were identified as protective factors (OR = 0.2) compared to the southern zone. Furthermore, molasses supplementation was identified as a protective factor (OR = 0.46), as was the presence of streams and creeks (OR = 0.56). The sale and purchase of cattle were also found to be protective factors (OR = 0.60; 0.42), as was the separation of animals (OR = 0.53). The minimization of the presence of visitors was also identified as a protective factor (OR = 0.59), as was the use of proper clothing (OR = 0.34) and the proper washing of clothing (OR = 0.59). Finally, the health registration of cattle was identified as a protective factor (OR = 0.63). Conversely, the presence of supplementation (OR = 9.44), a heated floor (OR = 1.69), access to a water supply (OR = 2.43), breeding or milk production of cattle (OR = 1.88), the storage of feed in storage or on the floor (OR = 1.5; 2.3), and the availability of technical or professional support staff (OR = 2.6; 1.6) were identified as risk factors (Table 4).

## 4. Discussion

This study aimed to clarify the epidemiological status of bovine neosporosis in dual-purpose livestock systems and identify the factors influencing its transmission dynamics. It was conducted in 150 farms across 24 municipalities in Huila, Colombia. The research involved sampling 360 cattle and collecting data on farm management and practices. The study highlights the key risk factors related to the exogenous transmission cycle of *Neospora caninum*, contributing to the development of targeted intervention strategies in the region.

The present study showed that 53% (191/360) of the tested animals were serologically positive for *N. caninum* antibodies. Similarly, Cruz-Estupiñán et al. (2019) obtained a prevalence of 52% (195/375) using the same ELISA technique [12]. However, the studies by Chaparro et al. (2014) and Oviedo et al. (2007) reported lower prevalences of 21.26% (84/397) and 10.2% (20/196) using indirect immunofluorescence and ELISA, respectively [7,13]. On the other hand, Villa et al. (2024) investigated the prevalence of *N. caninum* in milk serum by ELISA and found a seropositivity of 30.7% (180/586) [14]. On the other hand, Japa et al. (2019) investigated seropositivity by PCR detection of the DNA of the antigen in bovine placenta, obtaining a prevalence of 36.5% (35/96) [15].

Similarly, cattle in areas with temperate thermal soil have a higher risk of *N. caninum* infection (OR = 1.69). The pathogen requires a suitable environment for survival, and oocyst sporulation is temperature-dependent. For example, Rinaldi et al. (2005) mentioned that cattle reared at minimum temperatures in spring are at risk for neosporosis, and the same was found for areas with low vegetation cover in summer [16]. In addition, Schares et al. (2004) showed that the risk of *N. caninum* infection increases in areas with high temperatures close to the ambient temperature, favoring faster sporulation [17]. On the other hand, it is essential to note that oocyst survival is lower in hot and dry climates, suggesting that the epidemiology of the disease may vary seasonally [18]. Therefore, location in the central, northern, and western geographical zones was a protective factor compared to the southern zone because the latter has a climate of around 24–28 °C compared to the rest of the zones, which have an average of over 30 °C.

This study found no significant differences in age groups using binary logistic regression. However, the same rates of neosporosis were seen in these groups. Animals aged 106 months and older had a 60% prevalence (18/30). This suggests that disease is related to other factors or exposure to the pathogen, regardless of age. Nazir et al. (2013) found that 47% of animals over two years old had the disease, while 36% of those aged eight months to 2 years old and 39% of those under 8 months old had it [19]. Sadrebazzaz et al. (2004) also found no significant differences in the prevalence of *N. caninum* between age groups: 19.6% (49/250) of cattle in the 2–4-year-old group and 13.9% (53/382) in the 4-year-old group [20]. Selim et al. (2003) found that 2–4-year-old cattle and cattle >4 years old had different prevalence rates. The former had a prevalence of 32.38% (68/142), while the latter had a prevalence of 34.74% (66/124) [21]. However, Moore et al. (2014) found that older cattle are more likely to be infected at least once in their lifetime than younger cattle [22].

The study found that supplementation was a risk factor (OR = 9.44), but the reference population was very small (9/360). Bartels et al. (1999) found that poor-quality feed, such as moldy or leftover maize silage fodder, is a risk factor for *N. caninum*-associated abortion in the Netherlands [23]. Molasses is a supplement that should be stored correctly to correct nutritional deficiencies [24]. This is an essential factor for the prevalence of neosporosis, as found in our research (OR = 0.46). Storing food on the floor is a risk factor (OR = 2.3) because it is easy for dogs to access, as their feces can infect it, as are the conditions in which the food is stored. Similarly, Dijkstra et al. (2002) found that 92% of cattle farms with postnatal *N. caninum* seropositivity had evidence of canine defecation in feed aisles and storage places compared to 24% of the control group [25].

Drinking water from an aqueduct increases the risk of neosporosis (OR = 2.43), while drinking water from pipes and streams lowers the risk (OR = 0.56). Farmers usually store water from the aqueduct in tanks or drinking troughs, which can cause contamination and support the survival of *N. caninum*. Inadequate cleaning and not changing the water also spread the disease. Sierra et al. (2011) studied *N. caninum* in drinking troughs and storage wells on 15 cattle farms in Mexico, where 30 samples were collected and the antigen’s DNA was found in 90% of them [26]. Sadiq et al. (2023) found that most farms had cats and dogs sharing the food and water, which led to *N. caninum* contamination [27]. Similarly. Ould-Amrouche et al. (1999) found that using ponds is a significant risk factor for neosporosis (OR = 2.43) [28]. This is because ponds, like tanks or drinking troughs, have stagnant water.

Conversely, cattle that drink from streams and creeks are at a reduced risk of infection because these sources facilitate the dissemination of contamination from infected canine feces, thereby interrupting the life cycle of neosporosis. Azevedo et al. (2021) observed that 29.6% (68/324) of neosporosis-positive cattle consumed water from stagnant sources, which was identified as a risk factor (OR = 1.58) in comparison to flowing water, as evidenced by previous findings [29]. Nevertheless, Llano et al. (2018) examined the risk of neosporosis in cattle in Antioquia regarding open (non-stagnant) water sources and public (aqueduct) water sources and discovered no statistically significant discrepancy [30].

It is important to note that 83.3% of the animals were in holdings where the main activity was rearing and milk production. However, this type of production involves constant intervention and the personnel’s manipulation and handling of the cattle due to the different milking practices. In the same line, in our research, this activity presented a prevalence of 55.7% (167/300), while on farms with other cattle activities, the prevalence was found to be 40% (24/60). Breeding and dairy farming are also risk factors compared to other activities (OR = 1.88). López et al. (2007) obtained results in agreement with our research, where 39.9% (119/298) of cattle on farms whose main activity was dairy farming were seropositive for neosporosis, while only 2% (1/49) of cattle with other activities were seropositive [31]. Several studies on bovine neosporosis in Colombia have shown that this parasite is more common in dairy herds [32].

The sale of breeding animals is considered a protective factor (OR = 0.60), as is the purchase of breeding animals (OR = 0.42). This could be explained by the fact that the life cycle of *N. caninum* is interrupted by including or excluding animals. Frössling et al. (2005) concluded that no matter how low the prevalence of the disease is, it can persist for several generations by keeping positive heifers in production [33]. Therefore, selling and purchasing practices would help to renew the herd and, consequently, to eliminate the disease. In contrast, Oshiro et al. (2007) found that locally reared and purchased animals present risks of infection [34]. In the former case, the animals spend more time within the herd, which increases exposure to the pathogen; in the latter case, they may introduce the disease into the resident herd.

Farmers who segregate cattle on their farms are less likely to have neosporosis infection in their herd, so segregating animals by herd is a protective factor (OR = 0.53) because it reduces the number of cattle per herd, optimizes health management, and prevents horizontal transmission. Hassig and Gottstein (2002) found that flexible housing (non-separated herds) compared to traditional housing (separated herds) was a risk factor (OR = 9.17) for abortions due to *N. caninum* [35]. Similarly, Hobson et al. (2005) mentioned that housing heifers in split housing reduced the likelihood of abortion due to *N. caninum* because it was more difficult for dogs to access all areas [36].

This study shows that biosecurity protocols for employees and visitors can reduce cattle’s risk of neosporosis transmission. Having fewer visitors, washing clothes after contact with other animals, and wearing appropriate clothing were all found to help protect against infection. This is because hygiene and protocols on farms are essential. The disease can also be spread by things like clothing, equipment, or objects that have been contaminated [37]. Other studies have shown that farms should use biosecurity measures. Denis-Robichaud et al. (2019) found that 41% of farms had employees working with different farm animals. Only 49% of farms required the use of appropriate clothing. Farms rarely had visitor containment measures in place but always needed visitors to wear clean or disposable clothing [38]. Sarrazin et al. (2014) reported that 70% of farms in Belgium had boots and coveralls for entering the premises, yet only 20% were used [39]. Moore et al. (2010) found that 92.5% of farms had no-visitor signs, with 60% admitting more than 19 visitors per week and 12.5% having a visitor protocol [40].

Having a health record for each animal on the farm is considered a protective factor (OR = 0.63). This strengthens the farmer’s ability to provide comprehensive animal management and prevent disease by following the vaccination and worming schedule. In addition, recording health problems and conditions facilitates the early detection of pathologies. According to Calandra et al. (2014), it is essential to highlight clinical and epidemiological criteria to associate *N. caninum* infection with clinical signs [41]. When reproductive problems are recorded in cattle, the health record correlates with *N. caninum* infection, as clinical signs of abortion in cows characterize this disease (mummifications and embryonic resorptions), subfertility, and neonates with neurological signs (ataxia and paralysis) [42].

More research is needed on how technical or professional assistants spread *N. caninum* in cattle. The studies in the literature do not examine how management affects seropositivity, so we do not know much about this aspect of the disease. Trained cattle handlers are essential for preventing and treating diseases. Our study showed that on-farm professional support may be a risk factor (OR = 1.68). Giratá (2016) found some professionals do not know how to prevent, care for, and keep their farms safe from disease [43]. This lack of knowledge may help the parasite survive on farms. Davison et al. (1999) suggested taking preventive measures, including testing for the disease early on the farm [44]. Similarly, Peter et al. (2019) found that employees with lower education levels had worse husbandry practices than those with higher education levels [45]. Almeida et al. (2016) found that professional help reduced the risk of brucellosis (OR = 0.24) [46]. Oliveira et al. (2011) found that farms receiving professional assistance for ≤60 days were a risk factor (OR = 2.7) compared to farms receiving professional assistance for >60 days [47]. Almeida et al. (2016) found cases in which that veterinarians trained farmers [46]. Oliveira et al. (2011) concluded that their result was due to an increased risk of iatrogenic transmission [47].

## 5. Conclusions

Neosporosis is a little-known disease among farm workers in the region studied. A lack of information about the disease leads to inappropriate practices contributing to its spread. Insufficient technical or professional support was identified as a risk factor, as appropriate management measures are not always applied in livestock production. It is therefore essential to focus on the staff’s management of cattle on the farm to control the disease. Some practices, such as storing fodder on the ground or in inappropriate places or providing easily accessible water troughs for dogs, were found to be determinants of the occurrence of the disease. On the other hand, practices such as keeping animal health records, disinfecting people’s clothing, reducing contact with neighbors, and wearing different clothing for production, can help reduce the transmission of infectious diseases. It is essential to understand all the factors that influence neosporosis and to disseminate accurate information about the risk of transmission. This will help farmers to adopt appropriate practices and ultimately reduce the prevalence of the disease, thereby preventing economic losses to the sector.

## Figures and Tables

**Table 1:** Seroprevalence of neosporosis according to different age groups.

Age Groups (Months)	Frequence	Seroprevalence	95% Confidence Interval	
			Lower Limit	Upper Limit
12–45	27/59	45.8	33.0	58.4
46–65	67/115	58.3	49.3	67.3
66–85	62/124	50.0	41.1	58.9
86–105	14/24	58.3	38.5	78.1
≥106	18/30	60.0	42.5	77.5
Not received	3/8	37.5	4.0	71.0
χ^2^: 4.60; gl: 5; *p*: 0.466				

**Table 2:** Seroprevalence of neosporosis according to the different regions of area of Huila.

Region	Frequence	Seroprevalence	95% Confidence Interval	
			Lower Limit	Upper Limit
Northern	81/177	45.7	38.6	53.0
Western	29/60	48.3	35.6	61.0
Central	29/58	50.0	37.1	62.9
Southern	52/65	80.0	70.4	89.6
χ^2^: 23.48; gl: 3; *p*: < 0.001				

Source: own elaboration.

**Table 3:** Intervals for the use of dewormers with the corresponding seroprevalence.

Frequency of Deworming	Frequence	Seroprevalence	95% Confidence Interval	
			Lower Limit	Upper Limit
<2 months	18/29	62.1	44.5	79.7
Every 3 months	60/105	57.1	47.7	66.5
4 to 5 months	31/55	56.4	43.2	69.6
From 6 to 12 months	79/157	50.3	42.5	58.1
No reporting	3/14	21.4	0.2	43.0
χ^2^: 7.99; gl: 4; *p*: 0.092				

Source: own elaboration.

**Table 4:** Binary logistic regression analysis of disease determinants of neosporosis seroprevalence.

Variables		n	Positives	*p*	OR	95% Confidence Interval	
						Lower Limit	Upper Limit
Area conditions							
Geographical area	South	65	52	-	-	-	-
	Center	58	29	0.001 *	0.25 ***	0.11	0.55
	North	177	81	0.000 *	0.21 ***	0.18	0.41
	West	60	29	0.000 *	0.23 ***	0.11	0.52
Thermal flooring	Warm	218	104	-	-	-	-
	Temperate	140	85	0.016 *	1.69 **	1.10	2.61
Aqueducts	No	296	151	-	-	-	-
	Yes	64	45	0.003 *	2.43 **	1.36	4.36
Spouts and creeks	No	197	132	-	-	-	-
	Yes	163	40	0.008 *	0.56 ***	0.37	0.86
Animal nutrition							
Supplementing	Not	9	8	-	-	-	-
	Yes	351	190	0.035 *	9.44 **	1.17	76.29
Molasses supplementation	Not	281	121	-	-	-	-
	Yes	70	27	0.006 *	0.46 ***	0.28	0.80
Food storage	No	91	49	-	-	-	-
	Basket	133	63	0.986	1.00 **	0.59	1.71
	Storage	75	43	0.197	1.50 **	0.81	2.78
	Floor	46	31	0.027 *	2.30 **	1.099	4.84
Productive practices							
Breeding and milk production	No	60	24	-	-	-	-
	Yes	300	167	0.028 *	1.88 **	1.07	3.31
Sells bovines	No	149	60	-	-	-	-
	Yes	211	101	0.019 *	0.60 ***	0.39	0.92
Purchase bovine animals	No	274	116	-	-	-	-
	Yes	86	32	0.001 *	0.42 ***	0.26	0.71
Frequency of deworming (month)	From 1 to 2	29	18	-	-	-	-
	3	105	60	0.634	0.81 ***	0.35	1.89
	4 to 5	55	31	0.614	0.78 ***	0.31	1.98
	6–12	157	79	0.247	0.61 ***	0.27	1.39
Biosecurity measures							
Separating animals	No	93	34	-	-	-	-
	Yes	267	131	0.011 *	0.53 ***	0.32	0.86
Minimizing visitor presence	No	120	47	-	-	-	-
	Yes	240	117	0.021 *	0.59 ***	0.38	0.92
Use of appropriate clothing	No	109	33	-	-	-	-
	Yes	251	114	0.000 *	0.34 ***	0.21	0.56
Wash clothes properly	No	148	59	-	-	-	-
	Yes	210	101	0.018 *	0.59 ***	0.39	0.91
Records of the health of livestock	No	149	61	-	-	-	-
	Yes	210	102	0.037 *	0.63 ***	0.42	0.97
Support staff	N/A	90	52	-	-	-	-
	Professional	263	148	0.035 *	1.68 **	1.04	2.73
	Technician	6	4	0.281	2.61 **	0.45	15.02

* = statistical difference; ** = risk factor; *** = protective factor. Source: own elaboration.

## Data Availability

The data presented in this study are available on request from the corresponding author due to privacy reasons.
